# Effect of Penetration Enhancers and Safety on the Transdermal Delivery of Apremilast in Skin

**DOI:** 10.3390/pharmaceutics14051011

**Published:** 2022-05-07

**Authors:** Paulo Sarango-Granda, Lupe Carolina Espinoza, Natalia Díaz-Garrido, Helen Alvarado, María J. Rodríguez-Lagunas, Laura Baldomá, Ana Calpena

**Affiliations:** 1 Department of Pharmacy, Pharmaceutical Technology and Physical Chemistry, Faculty of Pharmacy and Food Sciences, University of Barcelona, 08028 Barcelona, Spain; paulogranda92@gmail.com (P.S.-G.); hl_alvarado@ub.edu (H.A.); anacalpena@ub.edu (A.C.); 2 Institute of Nanoscience and Nanotechnology (IN2UB), University of Barcelona, 08028 Barcelona, Spain; 3 Departamento de Química, Universidad Técnica Particular de Loja, Loja 1101608, Ecuador; 4 Department of Biochemistry and Physiology, Faculty of Pharmacy and Food Sciences, University of Barcelona, 08028 Barcelona, Spain; natalia.diaz.garrido@gmail.com (N.D.-G.); mjrodriguez@ub.edu (M.J.R.-L.); lbaldoma@ub.edu (L.B.); 5 Institute of Biomedicine of the University of Barcelona (IBUB), Sant Joan de Déu Research Institute, 08028 Barcelona, Spain; 6 Nutrition and Food Safety Research Institute (INSA-UB), 08921 Santa Coloma de Gramenet, Spain

**Keywords:** apremilast, squalene, skin, promoter

## Abstract

The poor water solubility of apremilast (APR) is the main impediment to the penetration of the drug through the skin barrier. The objective of this study was to evaluate the permeability of APR in different solutions enriched with penetration promoters in ex vivo samples of human skin, and additionally assess its tolerance in vivo. To this end, APR solutions with 5% promoter were developed, and the drug’s ability to penetrate human abdominal skin samples was evaluated; the coefficients of permeability, cumulated amounts permeated, and flow were some of the parameters evaluated; likewise, the in vitro and in vivo tolerance of the solutions was evaluated. The results obtained showed that the solutions containing squalene as a promoter improved the penetration of APR compared to the other promoters evaluated; in the same way, on an in vitro scale in HaCaT cells, the promoters were not toxic, finding a cell viability greater than 80% at the different dilutions evaluated. In the in vivo tests carried out with the solution that presented the best results (APR-Squalene solution), it was observed that it does not cause irritation or erythema on the skin after its colorimetric and histological evaluation of the dorsal region of rats after its application. Squalene becomes an excellent candidate to improve the permeability of the drug in the case of the development of a topical formulation; in addition, it was confirmed that this penetration enhancer is neither toxic nor irritating when in contact with the skin in in vivo tests.

## 1. Introduction

The transdermal drug administration consists of the application of a pharmaceutical product on the skin; the drug penetrates through the epidermis and the dermis and, in many cases, a dermal microcirculation can occur [1]. Transdermal administration provides a non-invasive alternative (unlike the parenteral route), minimizes the risk of toxic side effects, and avoids pre-systemic metabolism (oral route), improving bioavailability [2]. Pharmaceutical products, when administered to the skin, and upon contact and penetration through its layers, encounter dendritic cells (epidermis and dermis) that play a crucial role in immune responses [3]. There are some strategies to facilitate and enhance transdermal drug permeation including the use of penetration enhancers as well as laser and light devices in fractional mode [4].

The skin represents 16% of the total body mass (in an average person) and fulfills a protective barrier function. The skin allows the body to protect itself from the external environment and water loss [5,6]. The skin is divided into three sections, the epidermis, dermis, and hypodermis [7]. The possible routes of drug penetration through these layers of the skin are known as transepidermal and transappendageal routes [8]. The transepidermal route consists of the passage of the drug through the stratum corneum (a multicellular layer that is subdivided into several layers); this type of penetration can be intracellular (through corneocytes, keratinocytes, the transport of hydrophilic compounds prevails) or intercellular (transport of lipid compounds through intercellular spaces). The transappendegean route, on the other hand, consists of the passage of molecules through the hair follicles and sweat glands [9,10]

Penetration enhancers are substances that facilitate the transport of the drug through the skin; their properties include that they are mostly colorless and odorless substances, pharmacologically inert, specific in their mode of action, physically and chemically stable, non-toxic, non-irritating, non-allergenic, and have a reversible action [11]. Penetration enhancers act primarily on the stratum corneum and may influence drug diffusion through it or change partitioning in the stratum corneum; the most common penetration enhancers include fatty acids, alkanes, esters, terpenes, cyclodextrins, surfactants, and azone, among others [12,13].

Apremilast (APR) is a selective inhibitor of phosphodiesterase IV. The mechanism of action of this drug consists of modulating a series of proinflammatory and anti-inflammatory mediators (for example: TNFα, IL-23, IL-10, and IL-17), this modulation is achieved by increasing the levels of cyclic adenosine monophosphate (cAMP) intracellular [14]. APR is effective for the treatment of psoriasis, an autoimmune and chronic disease that is characterized by thick patches of inflamed, scaly skin due to an excessive proliferation of skin cells whose treatment is based on control of symptoms using phototherapy, and systemic and topical therapies with different drugs including corticosteroids, vitamin D3 analogues, methotrexate, acitretin, cyclosporine, and biological-targeted agents such as ustekinumab, secukinumab, and ixekizumab [15]. APR administration is currently approved, in the form of oral tablets of 10, 20, and 30 mg, manufactured and marketed by the Celgene Corporation under the trade name of Otezla, indicated for patients with psoriatic arthritis, and moderate to severe psoriasis, and patients with Behcet’s syndrome [16,17]. The effectiveness of this drug is limited by its side effects such as diarrhea, nausea, depression, and weight loss [18]. For this reason, the development of alternative administration forms, such as topical formulations, are attractive for local anti-inflammatory therapy; some research has covered this field with the development of nanocarriers incorporated into gels in some cases to improve the administration and bioavailability of APR [19,20,21,22,23].

The poor water solubility of APR is the main impediment to the penetration of the drug through the skin barrier (stratum corneum). Therefore, the objective of this study is based on evaluating the permeability of APR contained in a solution with different penetration promoters in ex vivo samples of human skin, and at the same time to evaluate their tolerance in vivo.

## 2. Materials and Methods

### 2.1. Materials

APR (purity: 99.6%; MW: 460.5 g/mol) was acquired from Wuhan Senwayer Century Chemical (Wuhan, China). Gattefossé (Barcelona, Spain) supplied Transcutol^®^ P [Diethylene glycol monoethyl ether]. The permeation promoters Azone^®^ [1-dodecylazacycloheptan-2-one] was supplied by Durham Pharmaceuticals (Durham, UK). Carene [3-Carene], Decanol [1-Decanol], Limonene [(S)-4-Isopropenyl-1-methyl cyclohexene], Menthone [(2S,5R)-2-Isopropyl-5-methyl cyclohexanone], Nonane [*n*-Nonane], Pinene [(+)-α-Pinene], and Squalene [2,6,10,15,19,23-Hexamethyltetracosane] were purchased from Sigma Aldrich (Madrid, Spain). Reagents for histological procedures were purchased from Sigma and Thermo Fisher Scientific (Barcelona, Spain). Reagents for cellular assays, HaCaT cell lines, and 3-[4,5-Dimethylthiazol-2-yl]-2,5- diphenyltetrazolium bromide (MTT) cell-proliferation assay were obtained from Gibco (Cacavelos, Portugal), Cell Line Services (Eppelheim, Germany), and Sigma (Barcelona, Spain), respectively. Ultrapure water was obtained from Water Millipore MilliQ purification system (Millipore Corporation, Burlington, VT, USA), and all the other chemical reagents used were analytical grade.

### 2.2. Validation of Analytical Method

An analytical method was validated by high performance liquid chromatography (HPLC) for the identification and quantification of APR. The HPLC system is detailed in previous studies [21].

A 200 µg/mL stock solution was prepared. Different calibration curves were prepared from the stock solution in two ranges and five concentrations of each. The injection volume for the low range was 50 µL and the high range was 20 µL:

Low range: 0.156, 0.313, 0.625, 1.25, 2.5 and 5 µg/mL.

High range: 5, 10, 20, 50, 75, and 100 µg/mL.

Data was collected and processed using Empower 3 software (Waters, Milford, CT, USA). Method validation was carried out in accordance with the International Conference on Harmonization (ICH) Q2A and ICH Q2B Guidelines [24]:

*Linearity*

The linearity of five calibration curves at two ranges, each with six concentration levels, was evaluated. The correlation coefficient (r^2^) obtained after the least squares linear regression analysis of the calibration lines was evaluated; in addition, the linearity ratios were evaluated using a one-way analysis of variance (ANOVA) test comparing the concentration of the standards. In relation to the areas obtained from them, this statistical treatment was carried out using the Graph Pad Prism^®^ 5.0 software.

*Accuracy and Precision*

Five calibration curves were performed on different days at two concentration levels: 0.156–5 µg/mL and 5–100 µg/mL. Accuracy was expressed as the percentage of relative error (%RE) (Equation (1)). Precision was defined as the relative standard deviation (*%RSD*) or correlation coefficient:

(1) $$\%\mathrm{RE}=\frac{C_{0}-C_{n}}{C_{n}}$$

where, *C*_0_ is the observed concentration; *C_n_* is the nominal concentration and %RE represents the mean percentage deviation (% relative error).

*Specificity*

The specificity of the method was evaluated by the absence of interference in the retention time of the analyte. Volumes of 20–50 µL were injected and the chromatographic profiles were analyzed at a wavelength of 230 nm. Five different samples were evaluated: (i) mobile phase blank, (ii) APR standard, (iii) skin blank as control, (iv) APR sample released through the skin, and (v) APR sample retained in skin.

*Sensitivity*

Sensitivity was measured by determining the limits of detection (LOD) and quantification (LOQ). The LOD and LOQ were measured as a function of the standard deviation of the response and the slope of the calibration curve. LOD and LOQ were calculated by:

(2) $$\mathrm{LOD}=\left[\frac{\left(3.3\;s\right)}{p}\right]$$

(3) $$\mathrm{LOD}=\left[\frac{\left(10\;s\right)}{p}\right]$$

where, *s* is the standard deviation of the Y-intercept, and *p* is the slope of the calibration curve.

### 2.3. Apremilast Solution Preparation

APR solution was prepared by dissolving APR in Transcutol^®^ P: water (60:40 *v*/*v*) and the addition of 5% *v*/*v* penetration enhancer (Table 1). A solution with a concentration of 1.5 mg/mL was obtained.

### 2.4. Ex Vivo Skin Permeation Test

The permeation of the drug through the skin was determined using the Franz diffusion technique [25]. Franz diffusion cells (Hanson Research, Chatsworth, CA, USA; Crown Glass Company, New Jersey, NJ, USA), with a diffusion area of 0.64 cm^2^ and receptor compartment of 6 mL, were used. Samples of abdominal human skin (Hospital de Barcelona, SCIAS, Barcelona, Spain) were used for this experiment in accordance with the Ethics Committee of the Hospital de Barcelona (17 January 2020). Human skin was dermatomized to 400 µm thickness with an Aesculap GA 630 dermatome (Aesculap, Tuttligen, Germany). The skin was equilibrated for 30 min and the integrity of the skin was evaluated based on its TEWL using Tewameter TM 300 (Courage & Khazaka Electronics GmbH, Cologne, Germany); those that met an acceptance criterion of TEWL less than 10 g m^−2^ h^−1^ were used [26]. The receptor medium consisted of Transcutol^®^ P: Water (60:40 *v*/*v*) in order to maintain *sink* conditions [27]. The assay was carried out at controlled temperature of 32 ± 0.5 °C and magnetic stirring of 500 r.p.m. Aliquots of 500 µL (APR-solutions 1.5 mg/mL) were added to the donor compartment of each cell. APR solution without promoter was used as a control (No promotor). Then, 200 µL aliquots were removed from the receptor compartment and replaced by the same volume of medium at different time intervals including 0 (pre sample time point), 14, 17, 20, 23, and 24 h. The samples obtained were quantified by HPLC as described in Section 2.2. Table 2 details the experimental conditions of the ex vivo permeation test. All solutions were made with skin from the same donor in triplicate, in this way an attempt is made to reduce the interindividual variability of the response due to biological differences.

### 2.5. Biopharmaceutical Parameter Data Analysis

The amount of drug permeated were determined by HPLC (Section 2.2). The permeation parameter such as: flux [*J_ss_* (µg/h^−1^/cm^−2^)] (Equation (4)), permeability coefficients [*K_p_* (cm^2^/h^−1^)] (Equation (5)), and cumulative permeated amount at 24 h [*Cum AP 24 h* (µg)] in skin were calculated per unit area as a function of the graph determined by the time of the test. The slope of linear portion was determinated using linear least-squares regression model with GraphPad Prism^®^ (GraphPad Software Inc. version 5.0, San Diego, CA, USA) Software.

(4) $$J_{ss}=\frac{Q_{t}}{A\cdot t}$$

where, *Q_t_* is the amount of drug that passed through the skin and was concentrated in the receptor compartment (µg); *A* is the area of the cell-cap for diffusion (cm^2^); and *t* is the time of the assay (h).

(5) $$K_{p}=\frac{J_{ss}}{C_{0}}$$

where, *J_ss_* is the flux calculated at the steady test and *C****_0_*** is the initial concentration of drug administer in the donor compartment.

The theoretical plasma concentration in human steady-state [*C_ss_* (ng/mL)] of the drug was used to predict the concentration of APR at the systemic level in a hypothetical surface of 25 cm^2^, it was obtained by Equation (6):

(6) $$C_{ss}=J_{ss}\cdot \frac{A}{Cl_{p}}$$

where, *A* is the hypothetical area of application and *Cl_p_* is the plasmatic clearance. The hypothetical area of application was 25 cm^2^. The plasmatic clearance value was 8.7 L/h [28].

### 2.6. Amount of Drug Retained in the Skin

The APR amount retained in the skin (Qret (µg/g skin/cm^2^)) was extracted in 1 mL of ACN by ultrasound water-bath technique. The skin samples were cleaned with gauze soaked in 0.05% dodecyl sulfate solution and washed with distilled water. The extract obtained was filtered and taken for analysis (Section 2.2).

### 2.7. In Vitro Skin Tolerance Study

To evaluate the effect of the different promoters that make up each APR solution on cell viability, the MTT assay was used in immortalized keratinocytes cell line HaCaT.

The cells were grown in Dubelcco’s Modified Eagle’s Medium (DMEM) -high glu-cose containing 25 mM HEPES, 1% non-essential amino acids, 100 U/mL penicillin, 100 g/mL streptomycin, and 10% heat inactivated Fetal Bovine Serum (FBS). Briefly, HaCaT cells were adjusted at 2 × 10^5^ cell/mL and seeded in 96-well plate and incubated by 48 h at 37 °C under 5% CO^2^ atmosphere until its adhesion. The experiments were performed with 80–90% confluency. Cells were incubated at different dilutions of the APR-solutions, these dilutions were from 1/50 to 1/2000 for 24 h. Untreated control cells were processed in parallel for comparison. Then, HaCaT cells were washed with PBS, incubated with MTT (2.5 mg/mL) for 2 h at 37 °C and processed as described previously [21].

Absorbance was measured at 570 nm in a microplate photometer Varioskan TM LUX (Thermo Scientific, Waltham, MS, USA). The percentage of cell survival relative to untreated control cells was calculated using the following equation:

(7) $$\%MTT=\frac{Absorbance\;of\;cells\;treated\;with\;compounds}{Absorbance\;of\;control\;cells\;without\;compounds}\;\cdot 100$$

### 2.8. In Vivo Skin Tolerance Study

The skin irritation potential of apremilast squalene solution was determined by a skin irritation test in rats [29]. This test was carried out with the approval of the Ethics Committee of the University of Barcelona and the Bellvitge Establishment, Barcelona, Spain (number 387/18, 26 November 2018).

Sprague Dawel rats (600–700 g) (*n* = 12) divided in three groups (*n* = 4) in a room with controlled temperature and humidity with food and water ad libitum, were used. The first group was treated with APR solution enriched with squalene (APR-Squalene solution), the second group was treated only with squalene solution without drug (Blank-Squalene solution), and the third group correspond to the positive control (Treatment with 0.8 mL of Xylol). The rats were shaved in the dorsal area 24 h before starting the test. Three areas were drawn in the dorsal region of the animal.

#### 2.8.1. Biomechanical Skin Properties Evaluation

Stratum corneum hydration (SCH) was measured in the basal state and ten minutes after the application of APR-Squalene and Blank-Squalene solutions in the treated area using a CM-825 Corneometer (Courage & Khazaka Electronics GmbH, Köln, Germany).

#### 2.8.2. Colorimetric Parameters

The possible changes at the skin level after the application of APR-Squalene solution and Blank-Squalene solution were evaluated through the color differences in the skin of the rat dorsal region with respect to the basal color, this assay was performed according to a study by Limon et al. 2017 [30] modified.

Skin color detection was performed on the dorsal area of the rats, using an MPA5 Multiprobe adapter with CL400 skin colorimeter probe from Courage + Khazaka electronic GmbH, Köln, Germany. The measuring probe on contact with the skin emitted a white LED light that homogeneously illuminated the area where it was applied; the light scattered on the skin is detected by the probe and expressed as light intensity: R, red; G, green; and B, white, on a numerical scale from 0 to 255 each.

A first measurement (basal value) was performed and then the addition of Xylol and APR-Squalene solution (or Blank-Squalene solution) was performed as indicated in Section 2.5.

The color determinations were performed 10 min after the application of the various solutions. The colors found were reproduced with the Microsoft Excel program from the RGB codes.

The treatment of the RGB color data obtained *A* (*R*_1_, *G*_1_, *B*_1_) and *B* (*R*_2_, *G*_2_, *B*_2_); the difference was obtained by calculating the linear distance in space between the two points and subsequently evaluating the distance; the following geometric equation was used for the linear distance:

(8) $$\left|\vec{AB}\right|=\sqrt{{\left(R_{2}-R_{1}\right)}^{2}+{\left(G_{2}-G_{1}\right)}^{2}+{\left(B_{2}-B_{1}\right)}^{2}}$$

From the results of the linear distance, the direction of the distance was determined through the determination of the general difference of the light intensity, using the following equation:

(9) $$\Delta_{intensity}=\left(R_{2}-R_{1}\right)+\left(G_{2}-G_{1}\right)+\left(B_{2}-B_{1}\right)$$

A negative result corresponds to a darker coloration, which is indicative of erythema and was assigned a value (+1), and a positive result corresponds to a lighter coloration, which indicates less erythema and was assigned a value (−1).

Finally, the difference between the two colors was obtained by multiplying the linear distance by the direction of the distance; the mean values obtained initially (basal) and those obtained after the induction of vasodilatation (application of xylol) were calculated and considered as 0% and 100%, respectively.

Consequently, the values obtained from the samples were calculated with respect to those obtained with 100% and in this way the sequence of the different stages of evolution of the erythema was traced.

#### 2.8.3. Histological Analysis

Rat skin biopsies were stored in cassettes for 24 h in 4% formaldehyde, the cassettes were washed by immersion in PBS (3 washes at 1 h intervals) and stored in 96% ethanol.

The samples were fixed in paraffin; sections of 6-µm thickness were stained with hematoxylin and eosin.

The samples were observed through a suitable Olympus BX41 microscope with an Olympus XC50 camera.

## 3. Results

### 3.1. Validation of Analythical Method

The conditions such as linearity, accuracy, precision, specificity, and sensitivity show that the method is specific for the detection and quantification of APR.

**Figure 1:**
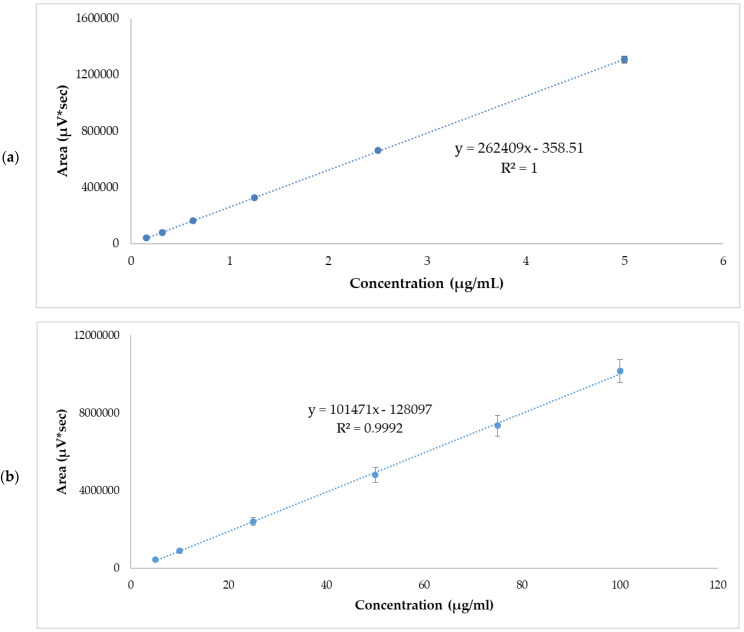
APR standard calibration curves. (**a**) Low range mean values, 0.156–5 µg/mL. (**b**) High range mean values, 5–100 µg/mL. Results are expressed as mean ± SD (*n* = 5).

Linearity was evaluated from five calibration curves at two concentration levels ranging from 0.156–5 µg/mL and 5–100 µg/mL. Table 3 and Table 4 show the areas obtained from each standard concentration. The r2 values of each of the calibration lines were >0.999. The graphic representation of mean values are shown in Figure 1. The statistical analysis of variance (ANOVA) showed that there were no statistically significant differences between the response areas obtained (*p* = 0.06 for low range and *p* *=* 0.39 for high range.

The accuracy and precision of the method were obtained through the analysis of samples with an APR standard concentration of 0.156–5 µg/mL and 5–100 µg/mL. The results are expressed as %RE and %RSD, for accuracy and precision, respectively. The data are reported in Table 5 and Table 6. These results show good precision with 11.93% and 13.28 for low and high range, respectively. The accuracy of the method was 2.54% and −12.21% for low and high range, respectively, for lowest standard concentration.

**Figure 2:**
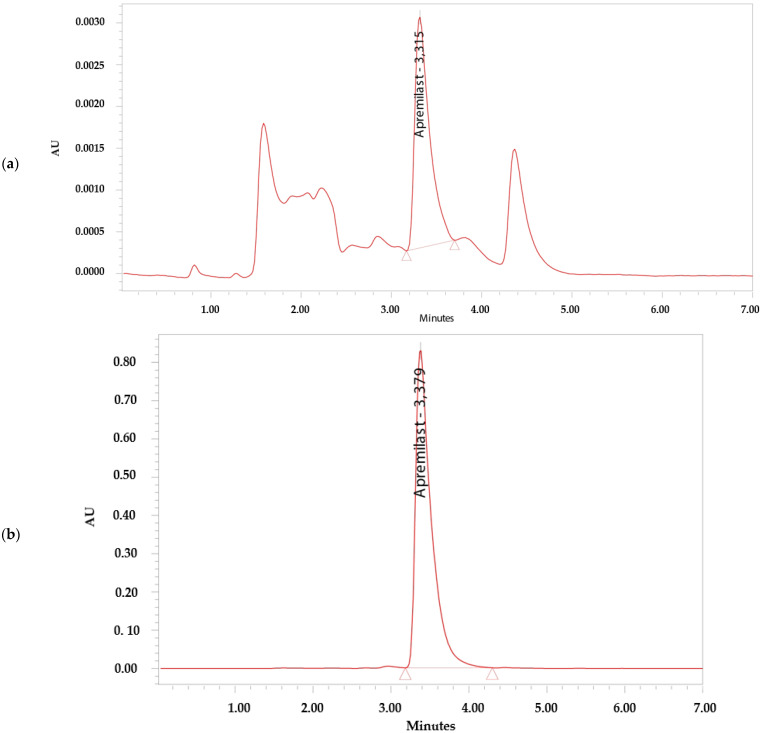

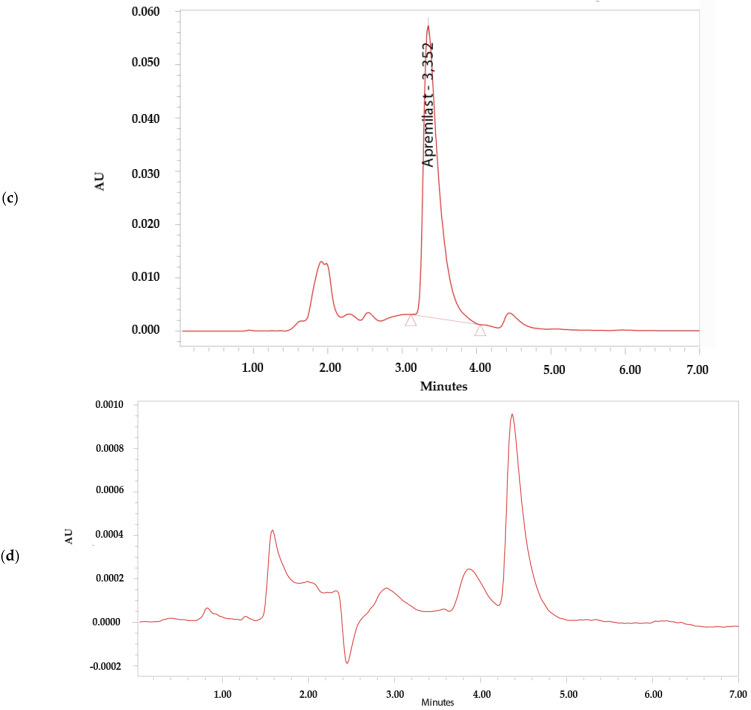
Chromatograms. (**a**) Apremilast standard 0.156 µg/mL. (**b**) Apremilast standard 100 µg/mL. (**c**) Apremilast extracted from human skin after the permeation study. (**d**) Blank sample.

The analytical method was considered specific because it demonstrated that there is no interference in the identification and retention time of APR (Figure 2). The retention time of APR was 3.3 min.

The LOD and LOQ were calculated using the response standard deviation and the slope of the calibration curve of 0.156–5 µg/mL and 5–100 µg/mL. From the flow and the Y-intersection of the five straight lines, the LOD for APR was established at 0.04 ± 0.05 µg/mL for low range and 5.70 ± 4.25 µg/mL for high range; the LOQ for APR was established at 0.11 ± 0.14 for low range and 17.28 ± 12.87 for high range. The method is sensitive enough for the drug determination.

### 3.2. Ex Vivo Skin Permeation Studies

The drug permeation was carried out in triplicate over a 24 h period using skin samples with normal TEWL values (10 g m^−2^ h^−1^) demonstrating skin integrity. The solutions showed slopes greater than 0.9, except the Menthone, which had a result of 0.86 in its linear section.

**Figure 3:**
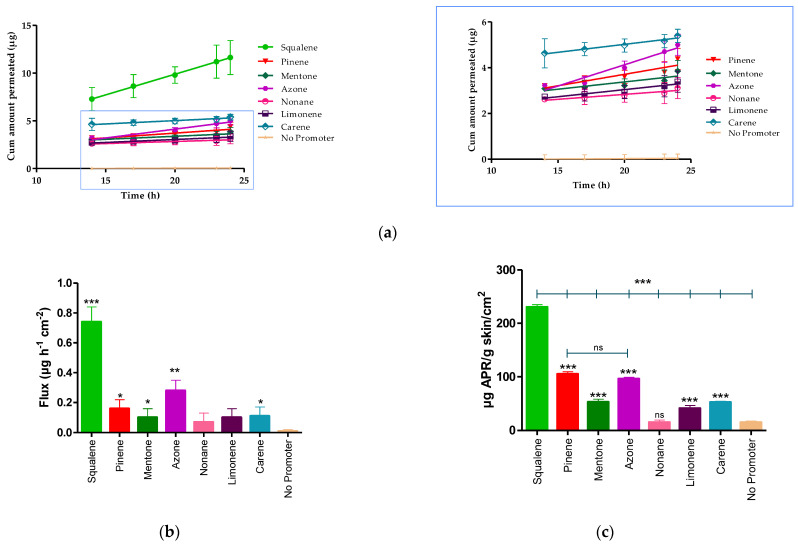
APR permeation profile. (**a**) Mean cumulative amount APR permeated. (**b**) APR-solutions flux. (**c**) APR retained amount in skin. Results are expressed as mean ± SD (*n* = 3). Statistically significant difference between solutions vs. No promoter: * = *p <* 0.05; ** = *p* < 0.01; *** = *p* < 0.0001.

The permeation profile and retained amount of APR are detailed in the Figure 3 and Table 7 shows the permeation parameters such as flux (*J_ss_*, µg/h^−1^/cm^−2^), permeability coefficient (*K_p_*, cm^2^/h^−1^), cumulative permeated amount 24 h (*Cum AP 24 h,* µg) and theoretical plasma concentration in human steady-state (*C_ss_,* ng/mL) of APR using various penetration enhancers.

The *Kp* is calculated from the flux dividing by the initial concentration of drug (1.5 mg/mL). After 24 h of assay, the amount of permeated drug (*Cum AP 24 h*) was 18.17 ± 3.10 μg for squalene. Statistical differences were found between the flux presented by squalene, pinene, mentone, azone, and carene compared to the APR solution without penetration enhancer (No promoter), whereas there were no statistically significant differences between nonane, and limonene compared to the solution without promoter.

The APR retained amount was more evident in the presence of squalene, pinene, and azone (Figure 3c) where squalene presented a greater amount retained (230.40 ± 4.50 µg/g skin/cm^2^), showing statistically significant differences with pinene (104.99 ± 4.30 µg/g skin/cm^2^) and azone (96.05 ± 3.10 µg/g skin/cm^2^).

### 3.3. In Vitro Tolerance Studies or Cell Viability Studies

**Figure 4:**
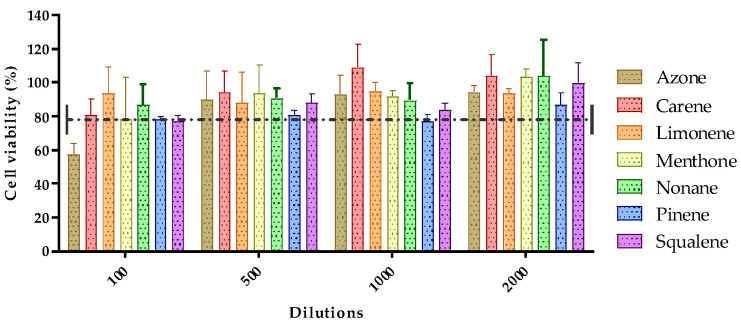
Effect of permeation promoters at different dilutions on the viability of HaCaT keratinocyte cells in vitro.

The toxicity of the seven permeation promoters used in this study was evaluated in skin cells (HaCaT cells) by the MTT assay. The results of treatment of HaCaT cells treated with different dilutions of the permeation promoters are shown in Figure 4.

Most of the permeation promoters tested were found not to induce cytotoxicity in the cells, except for azone which, at 1/100 dilution (highest concentration tested), induced cytotoxicity. The results suggested that the promoters evaluated do not generate skin irritation, but caution should be exercised with the use of azone; likewise, no correlation was found between the results obtained for skin cell toxicity with reference to its effect on drug penetration through the skin.

### 3.4. In Vivo Skin Tolerance Study

#### 3.4.1. Biomechanical Skin Properties Evaluation

Hydration in the stratum corneum was evaluated after the application of the two solutions (APR-Squalene solution and Blank-Squalene solution).

Topical application of these solutions on the skin significantly increased hydration in stratum corneum. These results were predictable, taking into account that the solutions are mostly made up of water and Transcutol.

**Figure 5:**
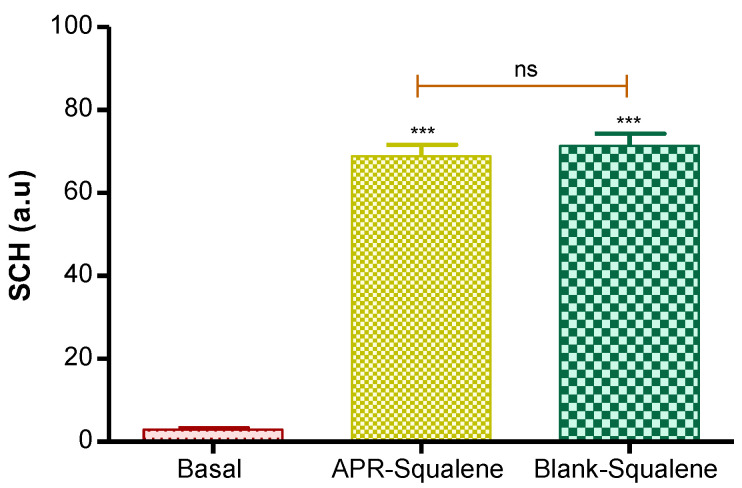
Stratum corneum hydration. *p* value: *** = p < 0.0001; ns = no significance.

These results suggest that the solutions with Squalene do not cause damage or irritation in the skin barrier (Figure 5).

#### 3.4.2. Colorimetric Parameters

The irritation potential of a solution containing APR and Squalene in its composition was evaluated. The irritation was measured through the color differences that appeared after the application of the solutions on the back of the rats’ skin.

The results were obtained as light intensity on a numerical scale for RGB and finally calculated with respect to the baseline value and expressed as % erythema.

Table 8 shows the values obtained from the colorimetric study for APR-Squalene solution and Blank-Squalene solution. It was evidenced that the APR-Squalene (4% erythema) and Blank-Squalene (3% erythema) solutions has not caused irritation after contact with the skin in comparison with the positive control (100% erythema).

To corroborate this information, an ANOVA was performed with the RGB values, and it was shown that the APR-Squalene and Blank-Squalene solutions presented statistically significant differences with a *p* value <0.0001, when compared with the positive control (Application of xylol as a skin irritant); on the other hand, it was evidenced that after the application of the solution on irritated skin it does not enhance the irritation.

**Figure 6:**
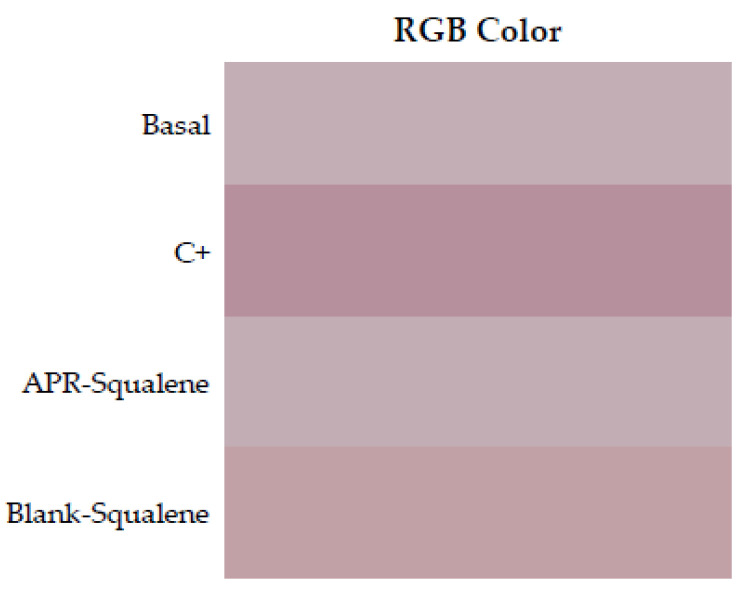
Erythema shown as skin color sequence section, using APR-Squalene solution and Blank-Squalene solution. Colors are reproduced from the Median values of RGB codes.

RGB colors were reproduced in a Microsoft Excel program. Skin redness was presented as a sign of irritation as shown in Figure 6 where the color reproduction marks greater redness after contact with xylol (C+) when it is compared to the RGB reproductions of the other groups. It is corroborated with the results showed in Table 8, which indicated with that the squalene and the combination of Squalene and APR are not irritating at the concentrations tested.

**Figure 7:**
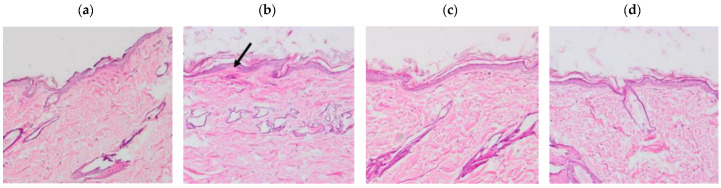
Histological sections of the dorsal area of rats (100× magnification). Arrows indicate signs of tissue damage. (**a**) Basal, (**b**) Xylol (C+), (**c**) APR-Squalene, (**d**) Blank-Squalene.

The results of the histological sections of the skin samples (Figure 7) showed tissue damage in those samples where xylol was applied, on the other hand, the samples where the solutions were applied do not show any damage.

## 4. Discussion

Unlike previous studies, the method was validated in a range of 0.156–5 µg/mL and verified from 5–100 µg/mL [21]. During ex vivo drug penetration studies, the components that make up the skin and excipients present in the formulations are released into the receiving fluid together with the active ingredient, so the matrix was evaluated to ensure correct identification and quantification of the drug.

Numerous methodologies have been described for the detection and quantification of APR [31,32,33]. However, the methodology described in this article was developed in a simple, easy way, with fewer chemical reagents and low cost, compared to the proposals of other authors [34,35]. In addition, a method under isocratic conditions is proposed, with a sample analysis time of 7 min and an APR retention time of 3.3 min. The objective of validating an analytical method is to demonstrate that it is adequate for the purpose for which it will be used; therefore, the analytical method of this study has been validated considering the implications that its use entails. The parameters of linearity, precision, accuracy, sensitivity, and selectivity have been determined according to the Harmonization Guidelines of the International Conference (ICH) [36] in the ranges of 0.156–5 µg/mL and 5–100 µg/mL, for the detection and quantification of APR from samples obtained from ex vivo permeation studies, using human skin as a matrix.

APR is insoluble in water [37] and most of the techniques described for the identification of active ingredients in complex samples require pretreatment. The receptor fluid used for in vitro permeation techniques consisted in transcutol:water solutions. Transcutol is commonly used in dermal formulations. Moreover, previous studies have reported the high solubilizing capacity of Transcutol for APR (Cs < 2.51~2.69 mg/mL) [21,38], and its use as part of the receptor fluid to guarantee the *sink* condition in the in vitro release and permeation studies [39].

The permeation of the drug through the skin was determined with the Franz Cell Diffusion technique and the membrane used was dermatomized human abdominal skin. The promoters evaluated have been extensively studied and were selected because there is research that has reported that these promoters increase drug penetration without having other pharmacological activities in the body. Moreover, they are non-toxic, non-irritating, non-allergenic, unidirectional, and compatible with drugs and excipients of dermal formulations, in addition to being cosmetologically acceptable [40,41]. However, despite its good performance in improving the skin permeability of drugs, there are still substances used as penetration enhancers that can cause skin irritation or, in some cases, skin toxicity in vitro in HACAT cells [42]. Azone, which was patented in 1976, is the first compound designed specifically as a penetration enhancer. It interacts with the structural lipids that make up the stratum corneum, thus allowing the passage of the drug, as well as acting by denaturing proteins and modifying the coefficient of diffusion of the drug favoring permeation through the skin [43,44]. The terpenes used in this study, such as Carene, Limonene, Mentona, Nonane, Alpha-pinene, and squalene, are organic compounds and laboratory designed that attract great interest. Terpenes are generally considered to be less toxic and have a potential of low irritation compared to other substances such as surfactants and other synthetic penetration enhancers [45]. Terpenes are a class of clinically acceptable and relatively safe permeation enhancers for hydrophilic and lipophilic drugs [40]. Terpenes are of natural origin and are generally considered “*safe*” by the US Federal Drug Administration, which allow them to offer advantages over other enhancers including alcohols, fatty acids, sulfoxides, and azone [46]. In this study, squalene was the most effective promoter in improving the permeability of APR through human abdominal skin. The squalenic acid chain confers high hydrophobicity for skin penetration of hydrophobic drugs, such as APR [47]. Squalene reduces the oxidative damage of free radicals in the skin; in addition, this promoter is present in a percent of around 13% in sebum which is produced by the sebaceous glands that correspond to small glands present in the skin that secrete sebum in hair follicles to lubricate skin and hair in animals [48]. Squalene is considered a great emollient in nature; it is quickly and efficiently absorbed into the skin [49]. Therefore, these results suggest that the incorporation of squalene in dermal formulations could be used as a strategy to improve the permeation of APR through the stratum corneum and their retention within the skin providing topical therapeutic alternatives with reduced side effects for the local treatment of psoriasis.

Squalene and the other penetration promoters evaluated in this study were non-toxic substances according to the results obtained for cell viability in HACAT keratinocyte cells. According to several studies, penetration promoters must be non-toxic or irritating substances, the absorption must be immediate and unidirectional [50,51]. In addition, after removing the material from the membrane, the tissue should immediately recover its barrier properties and the promoter should be compatible with the drug and pharmaceutical excipients [52].

For the in vivo tolerance test through animal models, APR solutions were used together with 5% squalene, as a penetration promoter. The evaluated colorimetric parameters showed that the squalene solutions (with and without drug) are neither irritant nor toxic when in contact with the back of the animal’s skin in comparison with the positive control and that was corroborated with histological tests. The histological results showed tissue damage after the application of xylol; however, this was not evidenced with the solutions evaluated. Consistent and reproducible color assessment is one of the most useful techniques in dermatology. Devices used for this purpose can quantify color, erythema, and tan in various skin types; the devices contain a spectrophotometer that analyzes the spectral characteristics of a color [53]. This technique is currently used due to its non-invasive nature and allows the characterization of injured skin from non-injured skin in patients with skin involvement; it is also used to evaluate the efficacy of drugs on skin pigmentation [54].

## 5. Conclusions

Squalene presented better results as an enhancer of the penetration of apremilast through the stratum corneum of a human abdominal skin sample, making it an excellent candidate to improve the permeability of the drug in the case of the development of a topical formulation. It was confirmed that squalene is a non-irritating, non-toxic, and non-allergenic substance that did not cause changes like irritation or erythema when in contact with the skin in experimental studies with animals.

## Figures and Tables

**Table 1:** Permeation enhancer used in this study.

Molecular Formula	Molecular Mass	Permeation Enhancer Substance	CAS Register Number
C_18_H_35_NO	281.5 g/mol	Azone^®^	59227-89-3
C_10_H_16_	136.2 g/mol	Carene	13466-78-9
C_10_H_16_	136.24 g/mol	Limonene	5989-27-5
C_10_H_18_O	154.25 g/mol	Menthone	14073-97-3
C_9_H_20_	128.25 g/mol	Nonane	111-84-2
C_10_H_16_	136.23 g/mol	Alpha-Pinene	80-56-8
C_30_H_62_	422.8 g/mol	Squalene	111-01-3

**Table 2:** Experimental conditions for Ex vivo skin permeation test.

Condition	Description
Receptor fluid:	Transcutol^®^ P: Water (60:40 *v*/*v*)
Cell volume:	6 mL
Diffusion area:	0.64 cm^2^
Membrane:	Human skin
Thickness:	400 µm
Replicates:	3 replicates
Temperature:	32 ± 0.5 °C
Stirring:	500 r.p.m.
Dose:	500 µL (1.5 mg/mL)
Sample volume:	200 µL
Sampling times:	0 (pre-sample time point), 14, 17, 20, 23 and 24 h

**Table 3:** Standard APR curve and respective area response factor. Low range.

Concentration (µg/mL)	Ratio 1	Ratio 2	Ratio 3	Ratio 4	Ratio 5
0.156	252,307.69	269,166.67	249,397.44	256,782.05	242,217.95
0.313	260,434.50	262,038.34	256,102.24	249,444.09	259,776.36
0.625	264,326.40	258,344.00	253,980.80	256,646.40	260,494.40
1.25	265,984.00	263,853.60	257,673.60	260,917.60	266,177.60
2.5	268,948.00	266,495.20	261,592.80	261,648.80	264,435.20
5	254,123.20	265,660.60	263,674.80	260,534.60	264,838.60

**Table 4:** Standard APR curve and respective area response factor. High range.

Concentration (µg/mL)	Ratio 1	Ratio 2	Ratio 3	Ratio 4	Ratio 5
5	109,557.800	74,592.400	82,219.400	99,394.400	84,412.600
10	106,052.700	73,916.800	93,962.200	93,504.300	85,729.500
25	107,647.960	86,377.760	91,944.640	100,525.680	97,117.120
50	103,422.940	87,307.680	105,285.220	90,515.760	95,526.080
75	104,525.933	95,189.893	87,206.813	100,035.547	103,939.733
100	104,007.780	93,685.270	104,641.200	107,957.790	97,844.260

**Table 5:** Accuracy and precision inter-day data for APR standard solutions. Low range.

Theoretical Conc. (µg/mL)	Calculated Conc. (µg/mL)	%RE	%RSD
0.156	0.15 ± 0.02	2.52	11.93
0.313	0.31 ± 0.01	1.49	4.38
0.625	0.62 ± 0.00	1.18	0.58
1.25	1.25 ± 0.01	−0.31	1.04
2.5	2.52 ± 0.05	−0.92	1.79
5	4.99 ± 0.02	0.22	0.47

**Table 6:** Accuracy and precision inter-day data for APR standard solutions. High range.

Theoretical Conc. (µg/mL)	Calculated Conc. (µg/mL)	%RE	%RSD
5	5.70 ± 0.76	−12.21	13.28
10	10.19 ± 0.60	−1.82	5.89
25	25.079 ± 0.66	−0.31	2.64
50	48.79 ± 3.17	2.47	6.50
75	73.86 ± 4.78	1.54	6.47
100	101.38 ± 2.90	−1.36	2.86

**Table 7:** Biopharmaceutical parameters of Apremilast-solutions with and without penetration enhancer.

*n* = 3	*J_ss_* (µg h^−1^ cm^−2^)	*K_p_* (cm^2^ h^−1^)	*Cum AP 24* h (µg)	*C_ss_* (ng/mL)	*Q_ret_* (µg/g skin/cm^2^)
Azone^®^	0.28 ± 0.07 ^e,g,h^	1.88 × 10^−4 e,g,h^	8.15 ± 0.75 ^h^	0.81 ± 0.01 ^a,c,e,h^	96.05 ± 3.10 ^b,c,e,h^
Carene	0.11 ± 0.06 ^g,h^	7.21 × 10^−5 g,h^	8.41 ± 0.50 ^h^	0.31 ± 0.05 ^h^	52.27 ± 2.10 ^h^
Limonene	0.10 ± 0.06 ^g^	6.49 × 10^−5 g^	5.30 ± 0.92 ^h^	0.28 ± 0.03 ^h^	40.91 ± 5.50 ^h^
Menthone	0.10 ± 0.06 ^g^	6.67 × 10^−5 g^	6.01 ± 0.80 ^h^	0.28 ± 0.05 ^a,e,h^	52.61 ± 5.60 ^a,e,h^
Nonane	0.07 ± 0.06 ^g^	4.48 × 10^−5 g^	4.85 ± 0.90 ^h^	0.19 ± 0.02 ^b,c,h^	15.13 ± 4.20 ^b,c^
Pinene	0.16 ± 0.06 ^g,h^	1.04 × 10^−4 g,h^	6.84 ± 0.80 ^h^	0.45 ± 0.02 ^d,a,e,c,b,h^	104.99 ± 4.30 ^b,c,d,e,h^
Squalene	0.74 ± 0.10 ^a,b,c,d,e,f,h^	4.93 × 10^−4 a,b,c,d,e,f,h^	18.17 ± 3.10 ^a,b,c,d,e,f,h^	2.13 ± 0.02 ^a,b,c,d,e,f,h^	230.40 ± 4.50 ^a,b,c,d,e,f,h^
No promoter	0.01 ± 0.01	3.8 × 10^−6^	0.08 ± 0.30	0.02 ± 0.01	15.14 ± 2.40

^a^ Azone; ^b^ Carene; ^c^ Limonene; ^d^ Menthone; ^e^ Nonane; ^f^ Pinene; ^g^ Squalene; ^h^ No promoter. Results are expressed by mean ± SD (*n* = 3). One-way Analysis of Variance (ANOVA) with Tukey’s Multiple Comparison Test were performed to assess the statistical significance between groups at (*p* < 0.05). *Underline value:* Promoter enhancer that presents a greater retained amount of drug in the skin.

**Table 8:** Skin color measurements expressed in RGB code of APR-Squalene and Blank-Squalene solutions. Measurements were made in the established quadrants of the dorsal area of the animal. RGB values represent median (min-max). ANOVA *p* value *≤* 0.0001 compared solutions vs. C+.

Group	RGB Value			Difference			Squares			Sum Square	Square Root of Sum	Normalized Dose	%Erythema
	R	G	B	dR	dG	dB	dR	dG	dB				
**Basal**	195	174	181	0.0	0.0	0.0	0.0	0.0	0.0	0.0	0.0	0.0	0%
	(195–196)	(174–175)	(180–181)										
**C+**	183	144	157	−12.0	−30.0	−24.0	144.0	900.0	576.0	1620.0	40.3	8.1	100%
	(182–183)	(144–144)	(157–158)										
**APR-Squalene**	195	173	180	0.0	−1.0	−1.0	0.0	1.0	1.0	2.0	1.4	0.3	4%
	(194–195)	(170–174)	(179–181)										
**Blank-Squalene**	193	161	166	−1.0	0.0	0.0	1.0	0.0	0.0	1.0	1.0	0.2	3%
	(193–194)	(161–163)	(166–166)										

## Data Availability

Data are contained within the article.
